# MR imaging of hepatocellular carcinoma: prospective intraindividual head-to-head comparison of the contrast agents gadoxetic acid and gadoteric acid

**DOI:** 10.1038/s41598-022-23397-1

**Published:** 2022-11-03

**Authors:** Federico Collettini, Aboelyazid Elkilany, Marta Della Seta, Ingo G. Steffen, Jasmin Maya Collettini, Tobias Penzkofer, Moritz Schmelzle, Timm Denecke

**Affiliations:** 1 Department of Diagnostic and Interventional Radiology, Charité-Universitätsmedizin Berlin, Corporate Member of Freie Universität Berlin, Humboldt-Universität zu Berlin, and Berlin Institute of Health, Berlin, Germany; 2 Berlin Institute of Health (BIH), Anna-Louisa-Karsch 2, 10178 Berlin, Germany; 3 Department of General, Visceral and Transplantation Surgery, Charité-Universitätsmedizin Berlin, Corporate Member of Freie Universität Berlin, Humboldt-Universität zu Berlin, and Berlin Institute of Health, Berlin, Germany; 4 Department of Diagnostic and Interventional Radiology, Leipzig University Hospital, Leipzig, Germany

**Keywords:** Liver cirrhosis, Hepatocellular carcinoma

## Abstract

The routine use of dynamic-contrast-enhanced MRI (DCE-MRI) of the liver using hepatocyte-specific contrast agent (HSCA) as the standard of care for the study of focal liver lesions is not widely accepted and opponents invoke the risk of a loss in near 100% specificity of extracellular contrast agents (ECA) and the need for prospective head-to-head comparative studies evaluating the diagnostic performance of both contrast agents. The Purpose of this prospective intraindividual study was to conduct a quantitative and qualitative head-to-head comparison of DCE-MRI using HSCA and ECA in patients with liver cirrhosis and HCC. Twenty-three patients with liver cirrhosis and proven HCC underwent two 3 T-MR examinations, one with ECA (gadoteric acid) and the other with HSCA (gadoxetic acid). Signal-to-noise ratio (SNR), contrast-to-noise ratio (CNR), wash-in, wash-out, image quality, artifacts, lesion conspicuity, and major imaging features of LI-RADS v2018 were evaluated. Wash-in and wash-out were significantly stronger with ECA compared to HSCA (P < 0.001 and 0.006, respectively). During the late arterial phase (LAP), CNR was significantly lower with ECA (P = 0.005), while SNR did not differ significantly (P = 0.39). In qualitative analysis, ECA produced a better overall image quality during the portal venous phase (PVP) and delayed phase (DP) compared to HSCA (P = 0.041 and 0.008), showed less artifacts in the LAP and PVP (P = 0.003 and 0.034) and a higher lesion conspicuity in the LAP and PVP (P = 0.004 and 0.037). There was no significant difference in overall image quality during the LAP (P = 1), in artifacts and lesion conspicuity during the DP (P = 0.078 and 0.073) or in the frequency of the three major LI-RADS v2018 imaging features. In conclusion, ECA provides superior contrast of HCC—especially hypervascular HCC lesions—in DCE-MR in terms of better perceptibility of early enhancement and a stronger washout.

## Introduction

Hepatocellular carcinoma (HCC) represents the fourth leading cause of cancer-related death worldwide and its incidence is increasing^1,2^. To empower curative treatments and thus attain long-term survival, unequivocal diagnosis at early stage of disease is critical^2^. Several organizations have published guidelines for the non-invasive diagnosis of HCC in patients at risk, including the American Association for the Study of Liver Diseases (AASLD), European Association for the Study of the Liver-European Organization for Research and Treatment of Cancer (EASL-EORTC) and the Asian-Pacific Association for the Study of the Liver (APASL)^3^. As non-invasive diagnostic criteria of HCC are essentially based on its vascular pattern—including contrast uptake during the arterial phase combined with the washout of contrast media during the portal venous or the delayed phases—these guidelines were established based on the assumption of dynamic computed tomography (CT) or magnetic resonance imaging (MRI) with extracellular contrast agents (ECA) being the first-line modality^3^. This diagnostic approach has been validated in several prospective studies sanctioning that, if this vascular pattern is confirmed, the diagnosis is established with almost 100% specificity^4^. However, in spite of an excellent specificity, the non-invasive diagnostic criteria show a relatively low sensitivity (50–60%) in nodules smaller than 2 cm^5,6^. This limitation prompted several groups to evaluate different strategies to improve diagnostic accuracy. The hepatocyte-specific contrast agent (HSCA) gadoxetic acid gadoxetic acid (Gd-EOB-DTPA, gadoxetate disodium; Primovist/Eovist, Bayer HealthCare, Berlin, Germany) combines the typical vascular behavior of gadolinium-based ECA with an early hepatocyte uptake starting already 60 s after administration and reaching a maximum plateau at 10–20 min^7^. Despite the fact that the use of HSCA resulted in significantly higher per-lesion sensitivity than MRI performed with ECA, non-invasive HCC diagnosis using HSCA does not reach the near 100% specificity displayed in most series with ECA^3^. The conviction that the decreased specificity may rely in the peculiar dual profile of HSCA has led several authors to argue that, until prospective studies with head-to-head comparison between ECA and HSCA have been carried out, non-invasive diagnosis of HCC using MRI should rely exclusively on extracellular contrast agents to assure the near absolute specificity^2^.

The aim of the present study was to conduct a quantitative and qualitative, intraindividual comparison of the MR-imaging features that define the typical vascular pattern of HCC on gadoxetic acid and gadoteric acid-enhanced MRI (gadoterate meglumine, Gd-DOTA; Dotarem, Guerbet) in patients with liver cirrhosis complicated by HCC.

## Results

The study included 23 patients (19 males and 4 females; mean age, 69 years; range, 53–87 years) with HCC and underlying liver cirrhosis. The etiologies for liver cirrhosis were as follows: hepatitis B virus induced cirrhosis (n = 3), hepatitis C virus induced cirrhosis (n = 4), alcoholic cirrhosis (n = 6), and cryptogenic cirrhosis (n = 10). The etiology of liver cirrhosis was diagnosed by hepatologists primarily based on clinical examination and laboratory parameters. Histopathological diagnosis (i.e., liver biopsy) was reserved for patients in whom definite diagnosis of the etiology of liver cirrhosis was not confirmed by the above-mentioned methods.

The mean longest diameter of the HCC lesions measured in the hepatobiliary phase (HBP) was 42 mm (range 17–124 mm). All HCCs were confirmed through histopathology either following surgical resection (n = 12) or preoperative using image-guided biopsy (n = 11).

### Quantitative analysis

**Figure 1:**
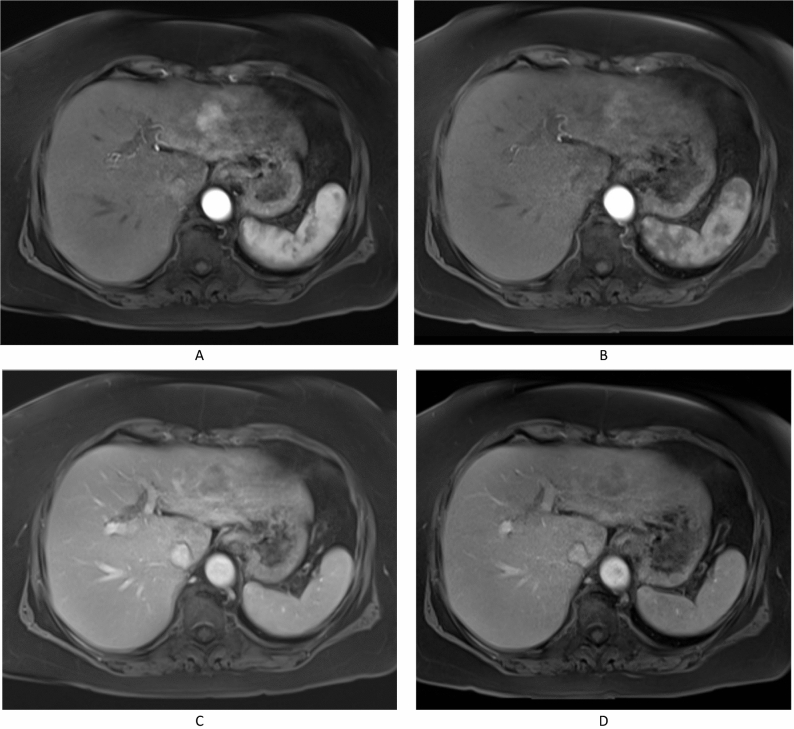
Wash-in and Wash-out dynamics of HCC lesions after administration of gadoteric acid and gadoxetic acid. Figures (**a,b**) demonstrates the stronger wash-in after administration of the extracellular contrast agent gadoteric acid (**a**) than with the hepatocyte-specific contrast agent gadoxetic acid (**b**). Figures (**c,d**) demonstrates the stronger wash-out following administration of gadoteric acid (**c**) than after gadoxetic acid (**d**).

Results of quantitative analysis are summarized in Table 1. During the late arterial phase (LAP), there was no significant difference regarding the signal-to-noise ratio (SNR) between gadoxetic acid-enhanced MRI (median of 298.7; IQR: 183.3–482.9) and gadoteric acid-enhanced MRI (median of 264.1; IQR: 220.3–380.4; P = 0.393). Contrast-to-noise ratio (CNR) was significantly higher with gadoteric acid-enhanced MRI compared to gadoxetic acid-enhanced MRI (median of 72.7; IQR: 50.8–165 vs median of 49.4; IQR: 18.1–154.4; P = 0.005). The wash-in of the HCC lesions was significantly higher (P < 0.001) after administration of gadoteric acid (0.9; IQR: 0.6–1.5) than after gadoxetic acid (0.4; IQR: 0.1–0.7). The wash-out of gadoteric acid was significantly stronger (Fig. 1) compared to that of gadoxetic acid (19.8; IQR: 7.3–35.6 vs. 9.3; IQR: −6.5 to 21; P = 0.006).

**Table 1:** Quantitative Analysis of signal-to-noise ratio (SNR), contrast-to-noise ratio (CNR), wash-in, and wash-out of HCC lesions in ECA-enhanced MRI and HSCA-enhanced.

	Extracellular contrast agent (ECA)		Hepatocyte-specific contrast agent (HSCA)		P value
	Median	IQR	Median	IQR	
SNR	298.7	183.3–482.9	264.1	220.3–380.4	0.393
CNR	72.7	50.8–165	49.4	18.1–154.4	0.005
Wash-in	0.9	0.6–1.5	0.4	0.1–0.7	< 0.001
Wash-out	19.8	7.3–35.6	9.3	−6.5 to 21	0.006

### Qualitative analysis

The results of qualitative analysis of overall image quality, artifacts and lesion conspicuity for both contrast agents and throughout the different perfusion phases are reported in Table 2.

**Table 2:** Qualitative analysis of overall image quality, artifacts, and lesion conspicuity for both contrast agents and throughout the different perfusion phases.

	Extracellular contrast agent (ECA)		Hepatocyte-specific contrast agent (HSCA)		P value
	Median	IQR	Median	IQR	
**Image quality**					
LAP	5	4–5	5	4–5	1.0
PVP	5	4–5	4	4–5	0.041
DP	4	4–5	4	3–5	0.008
**Artifacts**					
LAP	4	4–5	4	3–4	0.003
PVP	5	4–5	4	4–5	0.034
DP	4	4–5	4	4–5	0.078
**Lesion conspicuity**					
LAP	5	4–5	4	3–4.5	0.004
PVP	5	4–5	5	4–5	0.037
DP	5	4–5	5	4–5	0.073

*LAP* late arterial phase, *PVP* portal venous phase, *DP* delayed phase*.*

Overall image quality during the LAP did not demonstrate statistically significant differences between the two contrast agents (P = 1). During the portal venous phase (PVP) and delayed phase (DP), gadoteric acid-enhanced MRI showed a significantly higher overall image quality (P = 0.041 and 0.008, respectively).

**Figure 2:**
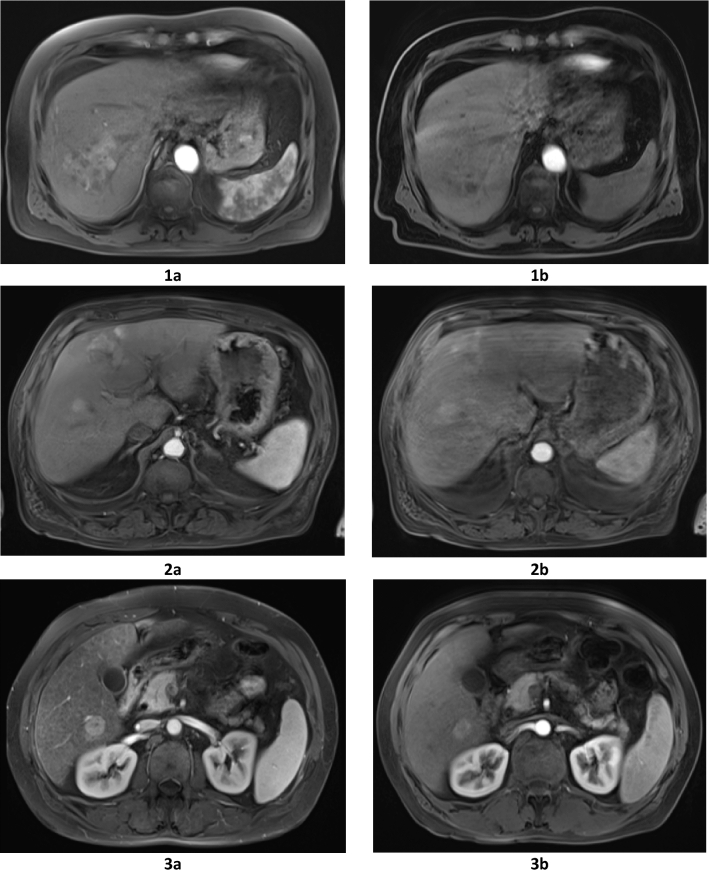
Qualitative analysis of overall image quality, the presence of artifacts as well as the conspicuity of HCC lesions during the arterial phase after administration of gadoteric acid and gadoxetic acid in three different patients. Figures(**1a–3a**) demonstrates higher image quality, lower artifacts as well as the higher conspicuity of HCC lesions during the arterial phase after administration of the extracellular contrast agent gadoteric acid than with the hepatocyte-specific contrast agent gadoxetic acid (**1b–3b**).

Artifacts where more frequent with gadoxetic acid-enhanced MRI. Gadoteric acid-enhanced MRI scored significantly higher grades during LAP and PVP (P = 0.003 and 0.034 respectively). During LAP, nine patients displayed moderate or severe artifacts after gadoxetic acid-enhanced MRI, while only one patient had moderate or severe artifacts when gadoteric acid was used (Fig. 2). The difference in the incidence of artifacts between the two contrast agents was not statistically significant during the DP (P = 0.078).

Lesion conspicuity was higher using gadoteric acid during both LAP and PVP (P = 0.004 and 0.037). During the LAP, excellent lesion demarcation was reported in 15 patients who underwent gadoteric acid-enhanced MRI and in six patients who received gadoxetic acid-enhanced MRI (Fig. 2). There was no significant difference in lesion conspicuity between the two contrast agents during the DP (P = 0.073).

### Major imaging features of LI-RADS v2018

There was no significant difference between both contrast agents regarding the frequency of the major imaging features of Liver Imaging Reporting and Data System (LI-RADS) v2018 (Table 3).

**Table 3:** Major imaging features of LI-RADS v2018 including arterial phase hyperenhancement, non-peripheral wash-out and enhancing capsule for both contrast agents and throughout the different perfusion phases.

	Extracellular contrast agent (ECA)	Hepatocyte-specific contrast agent (HSCA)	P value
**Arterial phase hyperenhancement**			
LAP	21/23 (91.3%)	17/23 (73.9%)	0.133
PVP	–	–	–
DP	–	–	–
**Non-peripheral washout**			
LAP	–	–	–
PVP	19/23 (82.6%)	17/23 (73.9%)	0.479
DP	21/23 (91.3%)	(20/23 (87%))	1.0
**Enhancing capsule**			
LAP	–	–	–
PVP	15/23 (65.2%)	10/23 (43.4%)	0.073
DP	17/23 (73.9%)	12/23 (52.1%)	0.073

*LAP* late arterial phase, *PVP* portal venous phase, *DP* delayed phase.

**Figure 3:**
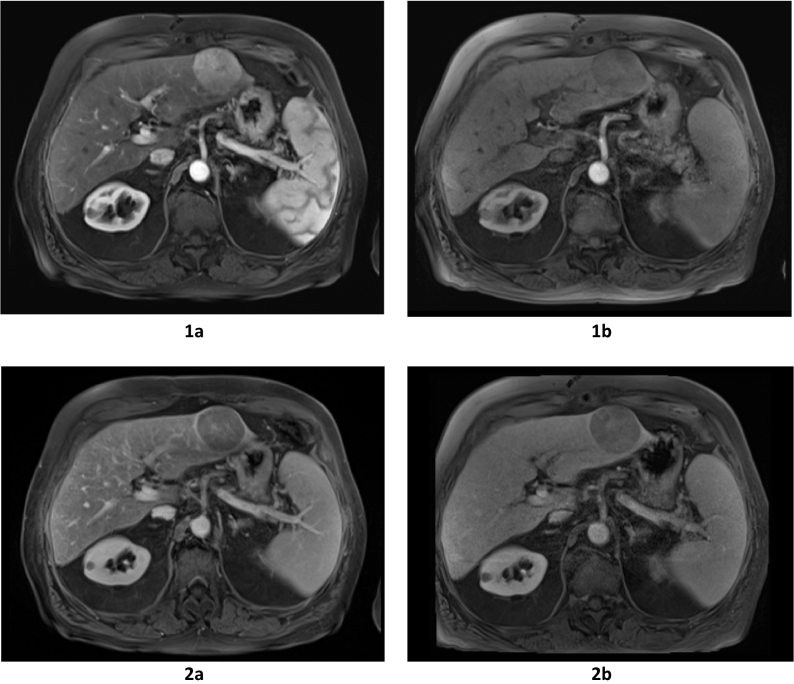
Major imaging features of LI-RADS v2018 (APHE and enhancing capsule) after administration of gadoteric acid and gadoxetic acid. Figures (**1a,2a**) demonstrates the stronger APHE during the arterial Phase (**1a**) and enhancing capsule in the portal venous phase (**2a**) after administration of the extracellular contrast agent gadoteric acid than with the hepatocyte-specific contrast agent gadoxetic acid (**1b,2b**). *APHE* arterial phase hyperenhancement, *PVP* portal venous phase, *LI-RADS* Liver Imaging Reporting and Data System.

The percentage of the nodules displaying *arterial phase hyperenhancement (APHE)* was 91.3% on gadoteric acid-enhanced MRI and 73.9% on gadoxetic acid-enhanced MRI (P = 0.133, Fig. 3). *Non-peripheral washout* on the PVP was observed in 82.6% of the nodules on gadoteric acid-enhanced MRI and in 73.9% of the nodules on gadoxetic acid-enhanced MRI (P = 0.479). 91.3% of the nodules displayed non-peripheral washout on the DP when gadoteric acid was used and 87% when gadoxetic acid was used (P = 1.0). During the PVP, an *enhancing capsule* (Fig. 3) was observed in 65.2% of the nodules on gadoteric acid-enhanced MRI and in 43.4% of the nodules on gadoxetic acid-enhanced MRI (P = 0.073). During the DP, the same feature were reported in 73.9% of the nodules on gadoteric acid-enhanced MRI and in 52.1% of the nodules on gadoxetic acid-enhanced MRI (P = 0.073).

### Interobserver agreement

The majority of features showed moderate interobserver agreement (Table 4). Substantial interobserver agreement was observed during the delayed phase regarding non-peripheral washout (0.646 [95% CI: 0.011–1] for gadoteric acid and 0.617 [95% CI: 0.133–1] for gadoxetic acid) and enhancing capsule (0.0.642 [95% CI: 0.247–1.0] for gadoteric acid and 0.649 [95% CI: 0.341–0.957] for gadoxetic acid), as well as for detection of arterial phase hyperenhancement during the late arterial phase of gadoteric acid-enhanced MRI (0.623 [95% CI: 0.16–1]).

**Table 4:** Inter-observer agreement for different qualitative parameters including analysis of overall image quality, artifacts, and lesion conspicuity as well as major imaging features of LI-RADS v2018 including arterial phase hyperenhancement, non-peripheral wash-out and enhancing capsule for both contrast agents and throughout the different perfusion phases.

	Extracellular contrast agent (ECA)					Hepatocyte-specific contrast agent (HSCA)				
	κ value	SE	95% CI		P value	κ value	SE	95% CI		P value
			Lower border	Upper border				Lower border	Upper border	
**Image quality/artifacts**										
LAP	0.401	0.178	0.052	0.750	0.035	0.326	0.142	0.048	0.604	0.018
PVP	0.474	0.158	0.164	0.784	0.004	0.498	0.155	0.194	0.802	0.000
DP	0.378	0.182	0.021	0.735	0.032	0.55	0.131	0.293	0.807	0.000
**Lesion conspicuity**										
LAP	0.559	0.169	0.228	0.890	0.000	0.642	0.122	0.403	0.881	0.000
PVP	0.308	0.173	−0.031	0.647	0.030	0.378	0.142	0.100	0.656	0.002
DP	0.596	0.181	0.241	0.951	0.000	0.496	0.162	0.178	0.814	0.000
**Arterial phase hyperenhancement**										
LAP	0.623	0.236	0.160	1.086	0.001	0.489	0.154	0.187	0.791	0.006
**Non-peripheral washout**										
PVP	0.311	0.239	−0.157	0.779	0.132	0.465	0.203	0.067	0.863	0.025
DP	0.646	0.324	0.011	1.281	0.001	0.617	0.247	0.133	1.101	0.003
**Enhancing capsule**										
PVP	0.457	0.186	0.092	0.822	0.026	0.457	0.186	0.092	0.822	0.026
DP	0.642	0.188	0.274	1.010	0.002	0.649	0.157	0.341	0.957	0.002

*LAP* late arterial phase, *PVP* portal venous phase, *DP* delayed phase.

## Discussion

Multi-step hepatocarcinogenesis is associated with a complex transformation of the intranodular blood supply, characterized by gradual reduction of the portal supply and a concomitant increase of the arterial supply, eventually resulting in an entirely arterial supply of the nodule in moderately differentiated HCCs^8^. The resulting vascular pattern characterized by arterial contrast uptake (wash-in) followed by wash-out in the venous phases allowed defining the non-invasive diagnostic criteria for HCC^4^. Endorsed by the AASLD and the EASL guidelines, non-invasive diagnostic criteria were established based on the assumption of dynamic CT or MRI with ECA being the first-line imaging modality and were validated in several prospective studies^5,9^.

Hepatocyte-specific contrast agents such as gadoxetic acid currently represent the standard of care for the study of focal liver lesions in many centers. Although large head-to-head comparative studies are still lacking, numerous studies comparing the diagnostic performance of ECA and HSCA have shown a higher sensitivity of HSCA-enhanced MRI, particularly in small HCC^10–12^. However, the routine use of HSCA is not widely accepted and opponents invoke the risk of a loss in specificity with HSCA-MRI and the need for prospective comparative studies evaluating the diagnostic performance of both contrast agents. In fact, some relevant technical aspects concerning the optimal use of HSCA for an appropriate evaluation of the early dynamic phases of MRI as done currently with ECA are not well established for HSCA^13^. The use of the already validated diagnostic criteria based on the recognition of arterial contrast uptake and wash-out may be impaired by the slower injection rate of small amount of contrast material as well as the hepatic parenchymal uptake of gadoxetic acid in the HBP which overlaps the delayed phase in gadoxetic acid-enhanced MRI^13^. For this reason, differences in the enhancement characteristics of ECA and HSCA during early dynamic phases play a particularly significant role in this particular subset of patients.

In the present study, CNR in the LAP was significantly higher for gadoteric acid-enhanced MRI compared to gadoxetic acid-enhanced MRI. Consistently, also ROI-based quantitative measurements of wash-in and wash-out in the HCC lesions were significantly stronger after administration of gadoteric acid compared to gadoxetic acid. Temada and colleagues compared the enhancement effect of gadoxetic acid and gadoteric acid in healthy volunteers and reported a lower arterial vascular and parenchymal enhancement for gadoxetic acid compared with gadoteric acid^14^. Similarly, Chen et al. retrospectively evaluated the differences in HCC enhancement pattern on DCI-MRI using gadoxetic acid and gadoteric acid in 34 patients with histopathologically proven HCC. The enhancing effect of HCC and image contrast between HCC and surrounding liver parenchyma during arterial phase were significantly lower when using HSCA compared with ECA^15^.

The LI-RADS has been developed as a tool for the standardized interpretation of liver imaging findings in patients who are at risk for HCC^16^. Major imaging features in LI-RADS v2018 (APHE, nonperipheral washout, enhancing capsule, size, and threshold growth) are used to categorize observations as LI-RADS 3 (intermediate probability of malignancy), LI-RADS 4 (probably HCC), and LI-RADS 5 (definite HCC)^17^. In the present study, no significant difference could be found in the major imaging features of LI-RADS v2018.

The percentage of HCC nodules displaying APHE was insignificantly higher on gadoteric acid-enhanced MRI than on gadoxetic acid-enhanced MRI. Interestingly, none of the two nodules without APHE on ECA-enhanced MRI showed APHE on gadoxetic acid-enhanced MRI, while four of the six nodules without APHE on gadoxetic acid-enhanced MRI demonstrated APHE on gadoteric acid-enhanced MRI. A similar observation has been reported by Min et al. reporting a higher rate of HCC displaying APHE on ECA-enhanced MRI compared to HSCA-enhanced MRI (96.8% vs 87.4%)^18^. This phenomenon is most likely due to the fact that the dose of HSCA is one fourth of that of ECA, resulting in a greater difficulty to highlight APHE during the late arterial phase. Consistently, lesion conspicuity was rated significantly higher using ECA during the LAP, despite the fact that overall image quality during the LAP did not demonstrate statistically significant differences between the two contrast agents.

Non-peripheral washout was more frequent using ECA-enhanced MRI, however the difference was insignificant. These results are in line with those obtained by Min et al. who detected non-peripheral washout in 80% of HCC lesions on ECA-enhanced MRI and in 77.9% of the lesions on HSCA-enhanced MRI, although the difference in this latest study is less pronounced than from our study^18^. The fact that washout is more frequently appreciated in the delayed phase than in the PVP is well known^19^. In fact, also in the present study, when considering hypointensity during the DP as washout also on HSCA-enhanced MRI, the difference between the two contrast agents was less pronounced. In fact, on HSCA-enhanced MRI early contrast uptake into the hepatocyte during the DP might exaggerate washout resulting in some studies in even significantly higher rates of (pseudo)washout. In a study recently published by Song et al., HSCA-enhanced MRI was superior to ECA-enhanced MRI in detecting washout when considering hypointensity in the DP as a modified washout appearance^20^. To prevent this unwanted phenomenon, that may have an impact on the specificity of this major feature, the LI-RADS limits the detection of washout to the PVP when HSCA are used.

The reporting of enhancing capsule was significantly higher on ECA-enhanced MRI than on HSCA-enhanced MRI. These results are in agreement with the results published by Song et al. reporting a capsule appearance in 75.3% of the nodules on ECA-enhanced MRI and in 50.7% of the nodules on HSCA-enhanced MRI^20^.

A further point of discussion in the comparative evaluation between both contrast agents is image quality. In several studies, the use of HSCA has been shown to be associated with an increased incidence of transient respiratory motion artefacts during the arterial phase that could potentially reduce diagnostic image quality. The occurrence of such artefacts has been reported in a wide range of patients with an incidence ranging from 2.4 to 18%^9^. In the present study, artifacts were more frequent with HSCA-enhanced MRI resulting in significantly lower diagnostic imaging quality during the LAP. Accordingly, lesion conspicuity in the LAP was rated higher at ECA-enhanced MRI. However, despite ECA-enhanced MRI displayed a lower incidence of artifacts and a higher lesion conspicuity during the LAP, the overall image quality during the LAP did not demonstrate statistically significant differences between the two contrast agents.

Both the statistical and spatial distribution of noise (the two important preconditions for noise measurement in SNR/CNR based on “two-region” approach) could be influenced by the use of phased-array surface coils as well as the application of reconstruction techniques such as parallel imaging^21^. In the present study, noise measurement in SNR/CNR was based on the standard deviation (SD) of the background noise instead of the more recent alternatives such as the “difference method” or "difference of images" (SNRdiff) technique^21–23^. Several parameters such as coil geometry, phase-encoding direction, and acceleration factor can influence the noise distribution in parallel imaging. As a result, determination of the noise intensity using SD of background noise in parallel imaging may over- or underestimation of SNR/CNR^21,24^. Nevertheless, we may argue that several other published studies evaluating parallel imaging also utilized the two-region approach (i.e. SD of background noise) for calculation of SNR^25–28^.

The present study was conducted in accordance with the LI-RADS v2018 compared to the study by Min et al.^18^—the largest to date study comparing ECA and HSCA in patients with HCC—and the study by Song et al.^20^ which were based on LI-RADS v2017 and LI-RADS v2014, respectively. LI-RADS v2018 was modified to achieve simplicity and concordance with the American Association for the Study of Liver Diseases (AASLD) criteria released in 2018 and the Organ Procurement and Transplantation Network (OPTN) criteria, which make the present study more consistent with the clinical algorithm for diagnosis of HCC^29^. Another advantage of the present study is the quantitative analysis of CNR, SNR, washin and washout.

Several limitations of the present study need to be acknowledged. First, this is a single-center prospective study including patients with established HCC diagnosis in liver cirrhosis with a clinical indication for pretherapeutic liver MRI prior to liver surgery. This inevitably introduces a selection bias and did not allow to expand the results to a screening population. The inclusion of only HCC lesions is not consistent with the diagnostic workflow in clinical practice as our patient cohort did not include other liver malignancies or cirrhosis-related benign nodules mimicking HCC. However, our focus was comparing ECA- and HSCA-based MRIs with regard to HCC diagnosis rather than the validation of HCC criteria in LI-RADS. Second, the relatively small cohort of patients poses a potential limitation. Third, the use of a predefined delay of 15 s after contrast agent injection during the two MRI also represents a limitation as HSCA is disadvantaged by its smaller volume. Forth, pretherapeutic liver MRI in the present study was conducted on patients who had planned surgery.

In conclusion, the comparison of the two gadolinium-based contrast agents gadoteric and gadoxetic acid showed that gadoteric acid provides superior contrast and conspicuity of HCC (especially hypervascular lesions) in DCE-MRI in terms of better perceptibility of early enhancement and a stronger washout, which are the imaging hallmarks of HCC. However, it should be noted that additional information that might be obtained by hepatobiliary phase imaging—a benefit provided exclusively by hepatocyte-specific contrast agents—are missed by using extracellular contrast agents for DCI-MRI of the liver.

## Materials and methods

This prospective intra-individual comparative study was performed in accordance with the Declaration of Helsinki and approved by the local ethics committee of Federal Institute for Drugs and Medical Devices. The study was registered at the European Union Drug Regulating Authorities Clinical Trials (EudraCT; registration date: 23.08.2013, register number: 2013-002409-75). Between July 2014 and April 2018, we enrolled adult patients with established diagnosis of HCC who were scheduled to undergo preoperative MRI examination prior to liver resection. All patients included in the study signed an informed consent before enrollment in the study.

### Inclusion and exclusion criteria

Adult patients (> 18 years of age) with liver cirrhosis and diagnosis of HCC based on histology or noninvasive HCC diagnostic criteria were evaluated for study inclusion if they had a clinical indication for MRI of the liver.

Patients were excluded if they were or have been suspected to be pregnant or breastfeeding; were scheduled for liver transplantation; had a previous systemic or locoregional therapy for the HCC or had contraindications to MR imaging or impaired renal function (glomerular filtration rate (GFR) < 30 mL/min). In patients with diagnosis based on noninvasive diagnostic criteria, histology obtained after resection or before ablation served as reference standard. Only patients with histology proven HCC who completed both contrast-enhanced examinations were included in the analysis.

### MR imaging and contrast agent administration

Patients underwent two MRI examinations, one with the ECA gadoteric acid and the other with the HSCA gadoxetic acid. The two MRI examinations were performed with a minimum time interval of 24 h to allow washout of the first contrast agent and a maximum time interval of 7 days to minimize potential for disease progression and thus warrant comparability of the two MRI examinations. The mean time interval between the two MRI examinations was 1.7 days.

MRI examinations were performed on a single 3 T MR system (Magnetom Skyra; Siemens Healthcare, Erlangen, Germany) using an 18-element body matrix coil and a 32-element spine coil. Imaging protocol included T2-weighted 2D sequences with and without fat saturation (FS) and T1-weighted unenhanced 2D sequences with and without FS (including in-/opposed-phase technique). Diffusion weighted imaging was acquired using respiratory-triggered single-shot echo planar imaging with a b-value of 50, 400 and 800 s/mm^2^.

T1-weighted 3D gradient-recalled echo (3D-GRE) sequences with FS were acquired with breath-hold technique before administration of contrast material as well as and during arterial, portal venous and venous phase, respectively. To obtain a precisely timed late arterial phase, a predefined delay of 15 s after contrast agent administration was used^30^. Portal venous phase and delayed phase images were acquired 50 s, 90 s and 120 s after contrast agent administration. Hepatobiliary phase images (HBP) were obtained 20 min after administration of gadoxetic acid.

An improved parallel acquisition technique (PAT) algorithm for volumetric imaging (CAIPIRINHA, Controlled Aliasing in Parallel Imaging Results in Higher Acceleration), modifying the k-space acquisition pattern and adapting image reconstruction correspondingly was applied. Imaging parameters are summarized in Table 5.

**Table 5:** MRI sequences and parameters.

MRI sequence	Orientation	TR (ms)	TE (ms)	Flip angle (°)	ST (mm)	MATRIX	Acquisition time (s)
T1WI Ultrafast 3D GRE FS (CAIPIRINHA-VIBE)	Axial	4.3	1.9	9	3.00	192 × 320	10 s
T2WI-HASTE (BH)	Axial	1400—1600	80–90	150	6.00	384 × 265	55
T2WI-TSE FS (RT)	Axial	3600–5400	100–102	150	6.00	488 × 235	120
DWI/ADC	Axial	6400	50	150	5.00	134 × 108	126

*TR/TE* repetition time**, ***TE* echo time**, **BH breath-hold**, ***RT* respiratory-triggered**, ***DWI* diffusion weighted image**, **IP/OP in-phase/opposed-phase, *VIBE* volumetric interpolated breath-hold sequence, *GRE* gradient-recalled echo, *FS* fat suppression, *CAIPIRINHA* controlled aliasing in parallel imaging results in higher acceleration, *HASTE* half-Fourier acquisition single-shot turbo spin-echo, *TSE* turbo spin echo.

Contrast agents were administered by intravenous bolus injection trough an antecubital vein at a rate of 2 mL/s with an automatic injector at a dose of 0.1 mmol/kg of body weight for gadoteric acid and at dose of 0.025 mmol/kg of body weight for gadoxetic acid. Following contrast agent injection, a saline flush of 20 ml at the same injection rate was administered.

### Image analysis

For every examination, dynamic T1-weighted MR images (pre-contrast, late arterial phase, portal venous phase, and delayed phase, as well as HBP in gadoxetic acid-enhanced MRI) were reviewed in an anonymized and randomized manner on a dedicated picture archiving and communications system (PACS; PathSpeed workstation; GE Healthcare). Image analysis comprised a quantitative and a qualitative assessment and was performed in consensus by two readers blinded to the patients’ medical history. The target HCC lesions were marked by the study coordinator and nodule diameter was measured as the longest diameter on HBP images on gadoxetic acid-enhanced MRI.

### Quantitative analysis

Quantitative analysis was performed using operator-defined regions of interest (ROIs) to determine the signal intensity (SI). A third reader placed identical ROIs in the tumor, in the normal liver parenchyma, in the paraspinal musculature as well as in the background region along the phase-encoding axis in both MRI examinations for each patient at comparable slice positions. The normalization was necessary because SI values in MRI do not represent absolute values but need to be interpreted relatively to reference regions.

In case of heterogeneous HCC lesions, solid and homogenous areas of the focal lesion were chosen for ROI placement, while areas of blood vessels, bile ducts, intratumoral necrosis and/or hemorrhage were excluded.

The following parameters were used for quantitative analysis:

*Signal-to-noise ratio (SNR)* was calculated as SI_HCC LAP_/SD_N_, where SI_HCC LAP_ is the signal intensity of the HCC lesion in the late arterial phase and SD_N_ is the standard deviation of the background noise in the same perfusion phase.

*Contrast-to-noise ratio (CNR)* was calculated as (SI_HCC LAP_ − SI_liver LAP_)/SD_N_, where SI_HCC LAP_ is the signal intensity of the lesion in the late arterial phase, SI_liver LAP_ the mean SI of the background liver during late arterial phase and SD_N_ is the standard deviation of the background noise in the same perfusion phase.

*Wash-in *was calculated as SI_LAP_ − SI_pre_, where SI_LAP_ is the signal intensity of the lesion in the late arterial phase and SI_pre_ the SI of the same lesion before contrast agent application. *Wash-out* was calculated as (SI_LAP_ − SI_PVP_)/SI_LAP_, where SI_LAP_ is the signal intensity of the lesion in the late arterial phase and SI_PVP_ the SI of the same lesion in the portal venous phase. The SI values of the HCC lesion were normalized in each phase using the SI value of the paraspinal musculature.

### Qualitative analysis

The overall image quality, the presence of artifacts as well as the conspicuity of the HCC lesions were rated throughout the different perfusion phases on a 5-point Likert scale (1—nondiagnostic; 2—poor, severe artifacts; 3—moderate, moderate artifacts; 4—good, minor artifacts; 5—excellent, no artifacts). Overall image quality of the different phases was assessed based on the definitions proposed by LI-RADS v2018^17^. Briefly, the *LAP* was defined by a strong enhancement of the hepatic artery and its branches, substantial enhancement of the portal vein, slight parenchymal enhancement, and absence of hepatic venous enhancement. The *PVP* was defined as the postcontrast phase in which portal veins are fully enhanced, the hepatic veins are enhanced by antegrade flow and the liver parenchyma is at peak enhancement. In the *DP* portal and hepatic veins as well as liver parenchyma are enhanced but less than in PVP.

Moreover, the frequency of three major imaging features of LI-RADS v2018 on images obtained using both contrast agents was assessed. These major features were: (1) APHE, (2) non-peripheral washout and (3) presence of enhancing capsule. *Arterial phase hyperenhancement* was defined as tumor hyperenhancement compared with the surrounding liver parenchyma on LAP**. ***Non-peripheral washout *was defined as tumor hypointensity compared with the surrounding liver parenchyma on PVP or DP on ECA-enhanced MRI and on PVP alone on HSCA-enhanced MRI. A partial or complete hyperintense rim surrounding the nodule on PVP and/or DP defined the term *enhancing capsule*. In case of discrepancies between the two readers, a consensus was reached through discussion.

### Statistical analysis

Statistical analysis was performed using R version 3.5.1 (The R Foundation for Statistical Computing). Due to small sample size, a non-parametric distribution of metric data was assumed. In consequence metric data are given as median and interquartile range (25–75th-percentiles) and the paired Wilcoxon signed-rank test was used. Categorical data were analyzed using contingency tables and exact McNemar-test (2 × 2) as well as McNemar-Bowker-test (> 2 categories). All tests were two-sided, and the level of significance was set to 0.05. The kappa (κ) test and 95% confidence intervals (CIs) were used to determine the inter-observer agreement. The results were interpreted as slight agreement for κ values of 0.01–0.20, fair agreement for 0.21–0.40, moderate agreement for 0.41–0.60, substantial agreement for 0.61–0.80, and excellent agreement for 0.81–0.99^31^.

## Data Availability

The datasets used and/or analyzed during the current study available from the corresponding author on reasonable request.
